# Dietary adequacy of adult surgical orthopaedic patients admitted to a teaching hospital in Zambia; a hospital-based cross-sectional study

**DOI:** 10.1186/s40795-018-0245-8

**Published:** 2018-11-08

**Authors:** Nixon Miyoba, Irene Ogada, Jonathan Mulenga

**Affiliations:** 1 Department of Food, Nutrition and Dietetics, Kenyatta University, Nairobi, Kenya; 2 Kitwe Central Hospital, Nutrition and Dietetics Unit, P.O.Box 20969, Kitwe, Zambia; 3 Department of Surgery, Kitwe Central Hospital, Kitwe, Zambia

**Keywords:** Adult surgical orthopaedic patients, Dietary adequacy, Recommended dietary intakes, Teaching hospital

## Abstract

**Background:**

Sub-optimal dietary intake among in-patients including those with orthopaedic injuries is one of the factors that contributes to malnutrition and poor clinical outcomes in hospitals. The purpose of this study was to determine adequacy of intake of selected nutrients among adult surgical orthopaedic patients on standard hospital diet.

**Methods:**

A hospital-based cross-sectional study involving 98 adult surgical orthopaedic patients aged 18–64 years and admitted in low cost wards was conducted. A 24 h dietary recall was used to record food intake in the previous 24 h. Adequacy of energy, protein, calcium, iron, zinc, dietary fibre, sodium, folic acid and vitamin C were determined using nutrition standards for adult inpatients in one state of Australia. Food frequency of consuming selected foods was assessed using standard 7-day food frequency questionnaires. Data was collected over a period of three months.

**Results:**

The findings on nutrient intake indicated that mean energy, protein, calcium, sodium, iron, zinc, folic acid, dietary fibre and vitamin C intake were all below the recommended values (1919Cal, 61.67 g, 160.05 mg, 222.91 mg, 10.19 mg, 2.55 mg, 165.98 μg, 20.09 g and 22.60 mg respectively). Further, 24.4%, 8.5%, 26.7%, 5.5% and 15.2% of the participants met the Recommended Dietary Intakes (RDIs) of energy, protein, iron, vitamin C and dietary fibre respectively. Hospital food contributed more than 60% of the total nutrient intake of energy, protein, folic acid and dietary fibre.

**Conclusion:**

In this study, the mean consumption intake for all the selected nutrients was below the recommended dietary intakes. Since sub-optimal dietary intake is associated with malnutrition and poor clinical outcomes, it is recommended that the hospital should provide diets that meet the nutrient demands of adult inpatients.

## Background

Orthopaedic conditions are physical injuries that affect the musculoskeletal system of an individual [1] Globally, hundreds of millions of people suffer from orthopaedic injuries [2]. In the United States of America, musculoskeletal injuries account for more than 20% of patient visits to health care facilities [3]. The most prevalent orthopaedic conditions in the world are those associated with sprains, dislocations and fractures [4]. A study conducted in India reported that fractures are the most common type (68.6%) of orthopaedic injuries [5].

Adequate dietary intake is a fundamental aspect of orthopaedic’s nutritional care and well-being [6]. Evidence suggests that orthopaedic patients have elevated requirements of calcium, zinc, vitamin C and vitamin D [7]. Apart from high requirements for micronutrients, orthopaedic patients also have increased demand for energy and proteins [6]. This is due to hypermetabolic reactions associated with the inflammatory response to injury. Hypermetabolic state may result in nutrient depletion and poor nutrition status through loss of lean body mass.

Orthopaedic patients might end up admitted in hospital where they become partially or totally dependent on hospital food for their nourishment. Hospital food plays a major role in the nutritional care of inpatients [8]. It has been observed that meal services in hospital can significantly influence clinical outcomes [9]. In order to meet nutrient requirements of inpatients, hospital food must be of good quality and provide all the nutrients required for a specific medical condition [10]. In addition, hospital menus must be prepared to meet the daily recommendations for inpatients [10].

As main health care providers, public hospitals in Zambia should provide nutritionally adequate meals to hospitalized patients [11]. In Zambia, there is scarcity of literature on hospital nutrition standards to guide menu preparation and most of the meals served to in-patients are monotonous [12]. Further there is limited literature in sub-Saharan Africa and in particular Zambia on dietary adequacy of adult surgical orthopaedic patients. It is against this background that the current study sought to assess adequacy of energy, protein, calcium, sodium, iron, zinc, folic acid, dietary fibre and vitamin C among patients on standard hospital diet.

## Methods

### Research design, period and location

This study adopted a cross-sectional analytical study design with quantitative methods in data collection, analysis and presentation. The design was appropriate for this study to test for associations between demographic characteristics and dietary intake [13]. The study was carried out from June to October 2016 at a teaching hospital in Lusaka province, Zambia. It is the largest public hospital and main referral center in the country with a catchment of approximately 2 million people [14, 15]. The hospital has a 1655 bed capacity and offers various specialist services to the public and is the primary trauma center in Lusaka [14, 16].

### Study population

The study targeted adult orthopaedic patients, 18–64 years admitted in low cost surgical wards on standard hospital diet. Patients admitted in low cost wards pay less for medical fees and receive fewer amenities compared to those in high cost wards [17]. Low cost services are subsisdized and are therefore affordable for patients of low socio-economic status. Apart from eating regular meals served by the hospital, some patients in low cost wards consume externally sourced food, either purchased from food outlets or brought in from home. Othopaedic patients constitute 20–40% of the total admissions in surgical wards [18]. Eligible to participate in this study were adult surgical orthopaedic inpatients 18–64 years, admitted to low cost wards, able to talk and on standard hospital diet for ≥3 days. The study excluded cognitively impaired patients, critically-ill patients and those who declined to participate.

### Sampling techniques and sample size

The teaching hospital was purposively sampled out of the four main referral hospitals in the country because it is the primary trauma center with a relatively higher number of adult surgical orthopaedic patients [16]. Low cost surgical wards (male and female) were also purposively sampled because that is where orthopaedic patients who eat regular hospital meals are admitted. Participants were drawn from all low cost (three male and two female) surgical wards. The nurses-in-charge of each of the selected low cost surgical wards were requested for the admission records from which a sampling frame was generated. Comprehensive sampling was adopted to include all participants who satisfied the inclusion criteria and consented to participate in the study [19, 20].

### Research instruments

The researcher-administered questionnaire and data collection procedures were pre-tested at a similar referral hospital in Zambia. The pre-test was done on a selected sample of 10 adult surgical orthopaedic patients with conditions similar to those of the main study. The pre-test participants were not included in the main study. The procedures employed in pre-testing the instruments were identical to those used in the main study. Validity was ensured by use of already validated tools such as the standard 24-h dietary and 7-day Food frequency questionnaires (FFQ) [21, 22]. The test-retest method was used to determine the reliability of the instruments during pretest. Data was collected twice during the pretest at an interval of 2 days from 10 participants. A test re-test correlation coefficient of 0.72 (CI: 0.61–0.82) was computed from the two sets of data and found to be adequate [23]. Information was gathered from relevant foodservice staff regarding snacks if any, the hospital menu, the methods used to prepare hospital meals, ingredients added to hospital food and daily recommended cut-offs used in the preparation of hospital meals.

### Data collection instruments and procedures

Guided by researcher-administered questionnaires, one time face-to-face interviews were conducted in the local language with each patient on the ward. Information on admission diagnosis and some socio-demographic characteristics of the patients were obtained from patients’ files and recorded in the questionnaires. Additional information on the socio-economic characteristics was sought by asking the patients. In order to establish the nutrient intake of the participants, the 24 h dietary recall was conducted on two different days within the week and over the weekend. Participants were first asked to list all the foods and beverages consumed in the past 24 h. The 24 h period was defined as starting at the time of waking up until the time the patients went to sleep at night. In case the participants had forgotten any foods and beverages from the list of items consumed, a list of these was also solicited. Starting with the first food item on the list, participants were probed for details of the food items in terms of type of food. In situations where the patients were not aware of the details of food items such as the one brought from home, visitors were requested to furnish information on food preparation and ingredients used. Ingredients that were used in the preparation of hospital food were obtained through the Key Informant Interview (KII). Food models, pictures and volume cups were used to estimate the quantities of food and beverages consumed. The standard procedures employed to collect 24 h recall data were as recommended by other studies [21, 22]. The food frequency data was collected by asking about the dietary intake of the patients over a 7 day period. Patients were requested to state how often they eat selected food stuffs belonging to the following food groups: (i) cereals, grains, roots and tubers (ii) legumes, pulses and nuts (iii) meat, meat products, milk, milk products and eggs (iv) vegetables (v) fruits (vi) beverages (vii) fats, oils and sugars.

### Statistical analyses

Data from the 24 h recall were entered and analyzed using NutriSurvey software (2005) to obtain amounts of energy, protein, calcium, sodium, dietary fibre, iron, zinc, folic acid and vitamin C. The intakes of patients on different days was averaged and comparison was made of mean intakes with recommended dietary intakes (RDIs) [2]. The RDIs used are the ones employed by the Department of Health in one state of Australia [10]. Food frequency questionnaire data were entered and analyzed using SPSS version 21.0. Descriptive statistics in terms of means, frequencies, percentages, median and standard deviations were generated for medical and demographic characteristics, nutrient intake and food frequency. A *p*-value of less than 0.05 was considered statistically significant. Content analysis was conducted to analyze qualitative data using thematic areas that were based on the study objectives. The interview with relevant foodservice staff were transcribed, coded and summarized according to the identified themes. Qualitative information was used to triangulate quantitative data collected using questionnaires.

## Results

### Demographic characteristics of the study participants

A total of 104 patients were eligible to participate, 100 consented to be interviewed, while 4 declined. Out of those who consented, data were missing for 2 patients. Therefore, a total of 98 patients had fully completed data and constituted the sample size. The mean age of the study participants was 36.4 ± 9.44 years (Table 1). The mean number of days spent in hospital since admission was 17.33 ± 10.91, ranging from 5 to 60 days.

**Table 1:** Demographic characteristics of the study participants

	*N* = 98	
Characteristic	n (%)	Mean ± SD
Age		
< 20	3 (3.1)	36.4 ± 9.44
20–29	21 (21.4)	
30–39	29 (39.8)	
40–49	25 (25.5)	
50–64	10 (10.2)	
Sex		
Male	62 (63.3)	
Female	36 (36.7)	
Marital status		
Married	79 (80.6)	
Unmarried	19 (19.4)	
No. of days spent in hospital since admission		
03–10	21 (21.4)	17.33 ± 10.91
11–16	36 (36.7)	
> 16	41 (41.8)	

### Type of orthopaedic conditions among the study participants

**Fig. 1:**
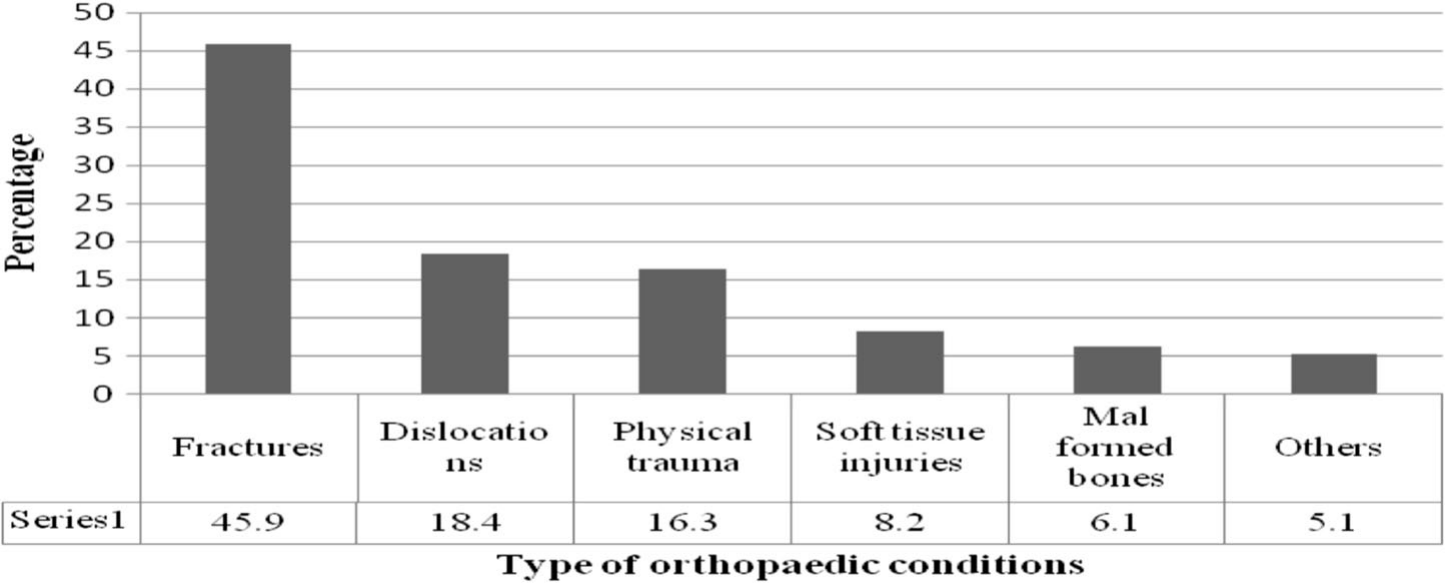
Type of orthopaedic conditions among the study participants

Most of the study participants (45.9%) had fractures, 18.4% had dislocations, while 16.3% had experienced physical trauma such as trauma of the leg (Fig. 1).

### Nutrient intake of patients based on the 24 h intake

The nutrient reference values used in this study are employed by the Department of Health in one state of Australia [10]. According to the reports, the standards are based on the Recommended Dietary Intakes (RDIs) and are reported to be appropriate for most acute patients in hospital. These reference values are also suitable for patients who are nutritionally well and those at risk of malnutrition. The standards relate to the following nutrients: energy, protein, fibre, vitamin C, folic acid, calcium, iron, zinc and sodium. Hospital menus that are based on these standards ensure that most patients meet their nutrient needs. However, patients with higher nutrient needs may require additional amounts of nutrients [24].

Mean energy consumption from total food intake was 1919.03 ± 364.70 cal per person (Table 2). Almost a quarter of the patients met the recommendation for energy intake.The mean consumption of protein was 61.67 ± 11.96 g per day, which was below the recommendation of 90 g per day and only 8.5% of the orthopaedic patients met the recommendation. The mean intake of all the selected micro-nutrients was below recommendations (Table 4.3). The mean intake of calcium was 160.05 ± 58.16 mg per day, while the mean intake of folic acid was 165.98 ± 59.32 μg per day. The mean intake of sodium was 222.91 ± 42.07 mg per day, while the mean intake of zinc was 2.55 ± 1.13 mg per day. Mean intake of iron and vitamin C among the orthopaedic patients was 10.19 ± 6.62 mg per day and 22.60 ± 12.36 mg per day respectively. None of the orthopaedic patients met the recommendations for micro-nutrients except for iron and vitamin C which was met by 26.7% and 5.5% of the participants respectively. The mean dietary fibre intake was below the recommendation of 30 g per day (20.09 ± 5.50 g/day) and only 15.2% of the patients met the recommendation.

**Table 2:** Nutrient intake of selected nutrients by the patients

Nutrient	^a^Recommended Dietary Intakes (RDI)	Total nutrient intake from both hospital and externally sourced food (mean ± SD)	Nutrient intake from hospital food (mean ± SD)	Nutrient intake from externally sourced food (mean ± SD)	Percentage contribution of hospital food	Percentage of participants with adequate intake
Energy (cal)	2200	1919.03 ± 364.70	1598 ± 449.54	321 ± 75.20	83.3	24.4
Protein (g)	90	61.67 ± 11.96	48.94 ± 14.68	12.73 ± 2.78	79.4	8.5
Calcium (mg)	1000	160.05 ± 58.16	0.27 ± 0.47	159.78 ± 38.35	0.002	0
Folic Acid (μg)	400	165.98 ± 59.32	126.25 ± 64.12	39.73 ± 9.65	76.1	0
Sodium (mg)	2300	222.91 ± 42.07	150.05 ± 54.86	72.86 ± 75.23	67.3	0
Zinc (mg)	14	2.55 ± 1.13	0.50 ± 0.23	2.05 ± 1.10	19.6	0
Iron (mg)	11	10.19 ± 6.62	2.08 ± 0.31	8.11 ± 5.03	20.4	26.7
Vitamin C (mg)	45	22.60 ± 12.36	8.5 ± 2.81	14.08 ± 5.69	37.6	5.5
Dietary fibre (g)	30	20.09 ± 5.50	16.33 ± 6.24	3.76 ± 0.49	80	15.2

^a^Agency for Clinical Innovation. Nutrition standards for adult inpatients in New South Wales Hospitals (2011)

The contribution of hospital food compared to externally sourced food was higher for some nutrients (energy, protein, folic acid, sodium and dietary fibre) apart from calcium, zinc, iron and vitamin C. This finding concurs with additional information from relevant foodservice staff which indicated that calcium-rich foods like milk and milk products are not served to inpatients. Further, iron-rich foods such as meat and meat products are only served once every fortnight. Moreover, fruits which are good sources of vitamin C are not served to orthopaedic patients by the hospital at all.

### Frequency of consumption of food items in the previous 7 days

#### Frequency of consumption of cereals, grains, roots and tubers

The starchy foods that were most consumed in hospital were maize nshima (stiff porridge) at 100% with a mean frequency of 7.08 ± 2.66, followed by bread at 36.7% (1.54 ± 2.30), rice at 18.4% (0.27 ± 0.68) and sweet potatoes consumed by 10.2% (0.18 ± 0.68) of the participants (Table 3). Fritters (a wheat flour dough product) were eaten by only 5.1% of the participants with a mean frequency of 0.10 ± 0.58. This finding concurs with the KII which found that maize nshima was the only cereal food served to orthopaedic patients on a daily basis.

**Table 3:** Mean frequency consumption of selected food items in the preceding 7 days

Food item	*N* = 98		
	Yes n(%)	No n(%)	Mean ± SD
Cereals, roots & tubers			
Maize nshima	98(100.0)	0 (0.0)	7.08 ± 2.66
Rice	18 (18.4)	80 (81.6)	0.27 ± 0.68
Sweet potatoes	10 (10.2)	88 (89.8)	0.18 ± 0.68
Bread	36 (36.7)	62 (63.3)	1.54 ± 2.30
Buns	6 (6.1)	92 (93.9)	0.21 ± 0.88
Fritters	5 (5.1)	93 (94.9)	0.10 ± 0.58
Legumes			
Beans	96 (98.0)	2 (2.0)	5.30 ± 1.83
Soy pieces	82 (83.7)	16 (16.3)	4.45 ± 1.01
Groundnuts	13 (13.3)	85 (86.7)	0.22 ± 0.60
Soy porridge	79 (80.6)	19 (19.4)	4.30 ± 2.44
Meat & dairy products			
Beef	22 (22.4)	76 (77.6)	0.42 ± 0.71
Chicken	17 (17.3)	81 (82.7)	0.31 ± 1.19
Fish	14 (14.3)	84 (85.7)	0.21 ± 0.56
Eggs	56 (57.1)	42 (42.9)	1.00 ± 1.02
Sausage	8 (8.2)	90 (91.8)	0.13 ± 0.47
Tea with milk	37 (37.8)	61 (62.2)	1.02 ± 1.49
Vegetables			
Chibwabwa (Pumpkin leaves)	7 (7.1)	91 (92.9)	0.12 ± 0.58
Kalembula(Sweet Potatoe leaves)	6 (6.1)	92 (93.9)	0.08 ± 0.34
Cabbage	42 (42.9)	56 (57.1)	1.10 ± 1.52
Rape	21 (21.4)	77 (78.6)	0.44 ± 1.00
Okra	7 (7.1)	91 (92.9)	0.12 ± 0.35
Fruits			
Oranges	45 (45.9)	52 (53.1)	1.84 ± 2.15
Apples	29 (29.6)	69 (70.4)	0.96 ± 1.86
Ripe bananas	61 (62.2)	37 (37.8)	2.34 ± 2.28
Beverages			
Tea with milk	37 (37.8)	61 (62.2)	1.02 ± 1.49
Carbonated drinks	22 (22.4)	76 (77.6)	0.62 ± 1.32
Other	25 (25.5)	73 (74.5)	0.40 ± 0.84
Fats & Oils	98(100.0)	0 (0.0)	7.08 ± 2.66
Sugar	44 (44.9)	54(55.1)	1.82 ± 1.99

#### Frequency of consumption of legumes, pulses and nuts

Almost all (98.0%) participants reported to have eaten beans in the last 7 days with a mean frequency of 5.30 ± 1.83 (Table 3). Soya pieces and soya porridge were consumed by 83.7% (4.45 ± 1.01) and 80.6% (4.30 ± 2.44) of the patients respectively. Groundnuts were consumed by 13.3% of the study participants with a mean frequency intake of 0.22 ± 0.60. The KII findings indicated that orthopaedic patients were served with soya porridge at breakfast on a daily basis and soya pieces on a fortnight basis.

#### Frequency of consumption of meat, meat products, eggs, fish and dairy products

Eggs were consumed by 57.1% of the participants with a mean frequency intake of 1.00 ± 1.02 (Table 3). Less than a quarter of the participants reported to have eaten beef (22.4%), chicken (17.3%) and fish (14.3%) with mean frequencies of 0.42 ± 0.71, 0.31 ± 1.19 and 0.21 ± 0.56 respectively. Only 8.2% of the participants reported to have eaten sausages with a mean frequency of 0.13 ± 0.47. Milk was consumed by 37.8% (1.02 ± 1.49) of the participants and was added to either tea or coffee. None of the study participants consumed plain milk. The KII revealed that beef is the only animal product that is provided by the hospital and is supplied irregularly because the hospital did not have a fixed menu.

#### Frequency of consumption of vegetables

Cabbage was eaten by 42.9% of the orthopaedic patients with a mean frequency of 1.10 ± 1.52 (Table 3). The three green leafy vegetables consumed by some participants in the last 7 days were chibwabwa (pumpkin leaves), kalembula (sweet potato leaves) and rape. Chibwabwa was consumed by 7.1% of the participants with a mean frequency of 0.12 ± 0.58. Only 6.1% of the study participants consumed kalembula with a mean frequency of 0.08 ± 0.34. Rape was eaten by 21.4% of the participants with a mean frequency of 0.44 ± 1.00. Okra was also consumed by few participants (7.1%) with a mean frequency of 0.12 ± 0.35. According to the KII findings, the only vegetable supplied by the hospital is cabbage and is served once or twice per week.

#### Frequency of consumption of fruits

Ripe bananas were the fruits most consumed (62.2%) with a mean frequency of 2.34 ± 2.28 (Table 3). With mean a frequency of 1.84 ± 2.15, oranges were eaten by 45.9% of the participants. Apples were the fruits least consumed at 29.6% with a mean frequency of 0.96 ± 1.86.

#### Frequency of consumption of beverages

The majority of the participants (37.8%) reported to have consumed tea with a mean frequency of 1.02 ± 1.49 (Table 3). Carbonated drinks were consumed by 22.4% of the patients with a mean frequency of 0.62 ± 1.32 in the last 7 days. Beverages are not served to adult surgical orthopaedic patients by the catering department.

#### Frequency of consumption of fats, oils and sugars

Sugar as part of rice or tea was consumed by 55.1% of the participants (Table 3). The mean frequency of sugar intake was 1.82 ± 1.99. All the patients (100%) reported to have consumed foods to which cooking oil or fat was added. The finding concurs with the KII which found that cooking oil is added to beans, cabbage, soya pieces and beef during preparation by the hospital.

### Additional information from relevant foodservice staff

When asked on how many meals are served to orthopaedic patients the Senior Catering Officer said “*Orthopaedic patients are served three main meals comprised of breakfast, lunch and supper*”. The respondent further explained that the hospital does not provide snacks to orthopaedic patients. The researcher asked the respondent on the ingredients added to hospital food. The response was “*cooking oil, tomatoes, onions and salt are added to cabbage, beans, soy pieces and beef during meal preparation*”. The respondent added that “*there is no document on nutrition standards at the hospital that should guide meal preparation*”. This was when asked if there was any document at the hospital to guide meal preparation. With regards the food service system used at the hospital, the catering officer explained that “*the hospital uses a centralized system whereby food is prepared in the main kitchen, held hot in covered buckets, plated and served as soon as possible to patients on wards*.”

## Discussion

### Demographic characteristics of the study participants

The finding that most of the study participants were male was not unique to this study. Other studies have reported similar findings. A study conducted among inpatients in Bangladesh, reported more male participants [25]. According to a cross-sectional study undertaken at the University Teaching Hospital (UTH) in Zambia, majority of trauma patients were male [16]. The possible reason for more male participants in the current study was because of a higher male population of inpatients in male wards compared to female ones. Further, orthopaedic injuries maybe more associated with males due to the higher likelihood of involvement in risky behaviour such as physical assaults and cycling on roads with heavy traffic [16].

The finding that majority of the participants in this study were of young age has also been reported in other studies. The mean age of the participants in this study is comparable to that reported in India [26]. A study at UTH reported that the mean age among participants with injuries was below 25 years [16].The variation in the mean age in various studies could be explained by the age composition of the participants. The study at UTH included both children and adults, while the current study only collected data from adult patients [16]. The majority of the participants in this study were married. Since the current study collected data from adults, it is expected that most of them would be married.

### Nutrient intake of the study participants

In this study, most patients did not meet their requirements for energy. The findings of this study are in agreement with those reported in other studies. In a study conducted in Britain, the mean intake of energy among orthopaedic patients was below recommendations [7]. Similar observations were made by two studies on osteoporotic patients done in Iran and Kenya [27, 28]. Effectiveness of treatment and recovery from disease are compromised if energy intake is insufficient among inpatients [10].

The present study observed that few orthopaedic patients met their daily protein requirements. Similar studies conducted among orthopaedic patients have also reported sub-optimal intake of protein [7, 28]. A study among women with osteoporosis also reported low protein intake [27]. The results of an Iranian study, however, reported adequate protein intake among osteoporotic patients [29]. In the present study, the mean protein intake was below the recommendation possibly due to inadequate intake of animal protein that supplies all the essential amino acids. This observation concurs with the results of the FFQ which revealed that the frequency of consuming meats and meat products was low, although a high frequency of beans and soya porridge was observed. High consumption of vegetable proteins such as beans among the patients only increased protein intake with incomplete amino acid profiles. Adequate protein intake is key in accelerating would healing, supporting growth and maintenance of body tissues.

This study has shown that the mean intakes for calcium, sodium, iron, zinc and dietary fibre were all below the recommendations. This finding is consistent with the results on inadequate intake of calcium, iron and zinc among orthopaedic patients [7]. Other studies conducted in Iran have also reported low intakes of calcium and zinc among osteoporotic patients [27, 29]. The intake of iron, was found to be higher than the recommended value [29].

According to the report on nutrition standards, the daily intake of sodium should not exceed the upper limit of 2300 mg per day [10]. There is need to be mindful that some illnesses such as renal disease require restriction of sodium [30]. High fibre foods should be offered in hospital to allow patients achieve an intake of 30 g per day of fibre [10]. Adequate intake of dietary fibre promotes the health of the digestive tract.

The low intakes of calcium, iron and zinc were expected in this study. This is because the FFQ revealed a low intake of foods rich in calcium, iron, zinc and other nutrients important for bone health [29]. For example, the frequency of consuming milk and milk products, meat and meat products, fruits and dark green vegetables was low. The low frequency of consuming milk and milk products and fruits was due to the fact that the hospital does not provide these food items to orthopaedic patients. At the teaching hospital, the only vegetable served to orthopaedic patients is cabbage and the frequency of intake in the preceeding 7 days was low. A study on dietary patterns of young children, suggests that high intake of dark green and yellow vegetables maybe positively associated with bone health [31].

The mean intake for vitamin C and folic acid among all the orthopaedic patients was below the daily recommendations. Hospital inpatients are usually deficient in vitamin C [10]. The finding of a study conducted in Iran revealed that the mean intake of vitamin C among osteoporotic patients was above the recommended daily allowances [27]. A possible reason for the low intake of vitamin C and folic acid in the current study was that the teaching hospital does not serve fruits and dark green vegetables to orthopaedic patients. This findings is supported by the low frequency (FFQ) of consuming fruits and dark green vegetables in the preceding 7 days. Evidence suggests that vitamin C plays a crucial role in collagen formation and wound healing in the human body [32]. Folic acid is important in the healing process of injuries through its action in amino acid metabolism, immune function and inflammatory response [33]. It also plays a significant role in red blood cell formation, which is usually very necessary after an injury, as in the case of orthopeadic patients who were the target of this study.

### Study limitations

The current study used the 24-h recall method and food frequency questionnaires both of which are based on the memory of the study participants. Despite not providing reliable estimates of usual intake, the 24-h recall is appropriate in resource-poor settings such as Zambia to measure intake of groups of subjects like orthopaedic patients [34]. The use of 24-h recall, food frequency questionnaires and interview with relevant foodservice staff helped to minimize recall bias. The other limitation of the study was the relatively small sample size. However, the use of a comprehensive sample helped to eliminate sampling errors by providing data on all the individuals in the population [20]. Comprehensive sampling techniques have been used before in other studies to collect data from small populations to include all participants [20]. Absence of African or Zambian Hospital reference standards was also a limitation in the current study. However, use of an Australian reference document helped to overcome the limitation.

## Conclusions

The findings indicate that the recommendations for all the selected nutrients (energy, protein, calcium, sodium, iron, zinc, folate, dietary fibre and vitamin C) were not met. Hospital food contributed over 60% of the total intake of energy, protein, folic acid and dietary fibre. Since sub-optimal dietary intake is associated with malnutrition and poor clinical outcomes, it is recommended that the teaching hospital should provide diets that help to meet the nutrient demands of adult inpatients. To build on the baseline information provided by the current study, a similar study on dietary intake should be conducted at the hospital using semi-weighed food records.

## Abbreviations

CI: Confidence interval
FFQ: Food frequency questionnaire
RDI: Recommended Dietary Intakes
SD: Standard deviation
SPSS: Statistical Package for Social Sciences
UTH: University Teaching Hospital
WHO: World Health Organization
